# Research Note: Comparison of *Enterococcus cecorum* genomes from broiler chickens with enterococcal spondylitis in Australian farms and strains from other countries

**DOI:** 10.1016/j.psj.2024.104356

**Published:** 2024-09-24

**Authors:** Delvin Otieno Combar, Soy Rubite, Peter C. Scott, Bronwyn E. Campbell, Thi Thu Hao Van

**Affiliations:** ⁎ School of Science, RMIT University, Bundoora West Campus, Bundoora, Victoria 3083, Australia; † Inghams Enterprises Pty Ltd, Somerville Victoria 3912 Australia; ‡ Scolexia Pty Ltd., Moonee Ponds, Victoria 3039, Australia

**Keywords:** spondylitis, *Enterococcus cecorum*, capsular polysaccharide, *cpsO* gene, whole genome sequencing (WGS)

## Abstract

Chickens in Australia have recently been identified with symptoms and morphological findings including spondylitis attributed to pathogenic *Enterococcus cecorum*. Notably, there is limited information on clinical *E. cecorum* strains in Australia. The *cpsO* gene, located downstream of the capsular polysaccharide *(cps*) locus, was recently reported to successfully differentiate between pathogenic and commensal *E. cecorum* strains, as this gene is highly conserved in the pathogenic strains. In this study, pathogenic *E. cecorum,* with a conserved *cpsO* gene, was detected on 1 of the 2 farms studied in Australia. *E. cecorum* strains isolated from clinical sites of the diseased birds from the second farm did not have the *cpsO* gene and were distant from the isolates of the first farm. A *cpsO* PCR of the caecal content of the birds on this farm was positive, while *cpsO* PCR of washed culture plates where the tissue extracts were spread onto and incubated for bacterial growth was negative. This suggests that pathogenic *E. cecorum* with the *cpsO* gene, as detected in Farm 1 and reported in other countries, was present in the second farm but could not grow on the selective agar plates during the initial step of *E. cecorum* isolation. Nevertheless, *E. cecorum* isolated from the clinical sites on the second farm might represent the pathogenic strain, but further animal studies are required to validate this possibility. Phylogenetic analysis showed that the pathogenic strains in Australia were most closely related to the clinical strains in North America.

## INTRODUCTION

Pathogenic *Enterococcus cecorum* causes conditions such as enterococcal spondylitis (ES), locomotor disorder, and septicemia in fast-growing broiler chickens (Dolka et al., 2016). ES causes deformity in the free thoracic vertebrae (FTV), resulting in the compression of the spine causing paralysis in severe cases. There is limited information on the pathogenic *E.cecorum* affecting broiler chickens in Australia, despite various studies on the pathogenic strain in other countries (Borst et al., 2015; Laurentie et al., 2023; Dolka et al., 2016). Therefore, understanding pathogenic *E.cecorum* and their virulence mechanisms would assist in reducing economic losses and improving animal welfare.

The pathogenic strains are characterised by putative virulence factors; enterococcal polysaccharide antigen (*ep*a) locus of *Enterococcus faecalis*, and capsular polysaccharide *(cps*) locus, similar to the capsule in *Enterococcus faecium* (Borst et al., 2015; Rhoads et al., 2024; Laurentie et al., 2023). The *cps* locus is responsible for resistance to neutrophil-mediated phagocytosis. An array of polysaccharide biosynthesis genes, encoded by an approximately 12kb region, exists as a downstream extension of this locus. The genes are highly conserved among pathogenic strains, but the commensal strains have variable (20–97%) identities, or the genes can be entirely absent (Borst et al., 2015). The *cpsO* gene, unique to pathogenic *E. cecorum* and located downstream from the *cps* locus, has been used for differentiation of pathogenic and commensal *E. cecorum* (Borst et al 2023).

This study aimed to isolate and sequence the genome of *E. cecorum* from Australian grown broiler chickens with spondylitis and compare them with strains reported from other countries. The study also investigated the presence of the *cpsO* gene in isolates from different tissues of the diseased birds.

## MATERIALS AND METHODS

Five birds from Farm 1 and 3 birds from Farm 2 were included in the study. They were 30-day-old Ross breed chickens from 2 free-range broiler farms in Mornington Peninsula, Victoria, with 30,000 birds per shed. The chickens showed signs of abnormality around the spine, demonstrating notable spondylitis symptoms. Animal ethics approval was not required as the samples were collected by the veterinarians in the course of their routine farm visits for veterinary care and surveillance. Tissue samples from clinical sites (vertebral lesions and leg joint, spleen, and caecal contents) were resuspended and diluted in 0.85% saline. One hundred microliters of each dilution were spread on Columbia agar (ThermoFisher Scientific) with 5% horse blood (Serum Australia) and 1.5% Nalidixic Acid and incubated in CO_2_-enriched conditions at 37°C overnight (Huang et al., 2023). After incubation, 4-5 colonies from each plate were subcultured onto Horse Blood Agar (HBA) plates (prepared as described above, except for the antibiotic). One hundred and ninety 2 isolates were obtained from vertebral lesions, joints, spleen, and ceacum samples.

A Bruker MALDI-TOF Microflex LT instrument (MALDI-TOF) was used to confirm the identity of the different colonies on the HBA. Selected colonies were transferred to a target plate, treated with matrix solution, and analysed to compare their spectra against the reference database for species identification. *E. cecorum* colonies were dissolved in Heart Brain Infusion media (HBI, ThermoFisher Scientific), grown overnight, and then stored in 30% glycerol at -80°C for future use.

The DNeasy PowerSoil Pro Kit (Qiagen) was used to isolate genomic DNA from bacterial colonies according to the manufacturer's instructions. End-point PCR was conducted using *cpsO*-specific primers (Forward: GCGATTGTTCCAAAGGTGTTAG; Reverse: AGTTTGAATGGCAAAGCTAATTC) to confirm the presence of a 195bp *cpsO* gene fragment (Borst et al., 2023) of the *E. cecorum* isolates obtained in this study and also the extracted DNA of the caecal content of all the birds studied. The PCR protocol for denaturation, annealing, and extension temperatures was 95°C for 1 min, 95°C for 15 min, 52°C for 15 min, 72°C for 15 min, and 72°C for 15 min with a total of 35 cycles respectively. DNA extracted from all the *E. cecorum* isolates, the caecal content of all the birds, and the washed plates where the tissues were spread onto and incubated for bacterial growth, were assessed for the presence of *cpsO*.

Out of the192 strains isolated, 68 were identified as *E. cecorum* by MALDI-TOF and were subjected to *cpsO*-PCR. Selected *cpsO*-PCR positive and *cpsO*-PCR negative isolates (13 in total) were subjected to whole genome sequencing. The genome library preparation was performed using the Nextera XT DNA Library Preparation Kit as directed by the manufacturer, and an Illumina Miseq was used to sequence the genomic DNA using a 2×300 bp (v3, 600 cycle) kit. A5-miseq was used to assemble the microbial genomes from Illumina Miseq data, using default settings for quality filtering, error correction, and scaffold construction. (Coil et al., 2014). The assembled genomes were uploaded to Rapid Annotation using Subsystems Technology (**RAST**) servers for annotation (Aziz et al., 2008). Genome sequences of *E. cecorum* isolates obtained in this study have been deposited at GenBank and can be accessed using the BioProject ID PRJNA1118070.

Phylogenetic analysis was conducted on previously studied strains and the isolated strains to understand the evolution of the different *E. cecorum* strains in Australia, using the Genome BLAST Distance Phylogeny method on the Type Strain Genome Server (Meier-Kolthoff & Göker, 2019; Meier-Kolthoff et al., 2022). Graphical images of the resulting trees were produced using the online Interactive Tree of Life program (https://itol.embl.de/). A total of 22 strains were used for the analysis.

## RESULTS AND DISCUSSION

*E. cecorum* was isolated from vertebral lesions, joints, spleen, and ceacum samples. Whole genome sequencing of the isolates showed that their genome sizes ranged from 2.0 to 2.3 Mbp, coding sequences (**CDS**) ranged from 2,150 to 2,406, and the GC content ranged from 36.2% to 36.7%, which was similar to that of *E. cecorum* strains reported in other countries (Table 1). Strains that were *cpsO*-PCR positive were isolated from 3 out of 5 birds on Farm 1, from vertebral lesion and leg joint samples, but no isolates from the caecum and spleen were *cpsO*-PCR positive. Commensal strains are generally isolated from the intestines, whereas pathogenic strains are isolated from extraintestinal organs (Borst et al., 2023). The pathogenic strains may also originate from intestines but they have the opportunistic capability of becoming systemic. Interestingly, for bird 5 in Farm 1, 2 different strains of *E. cecorum* were isolated from the joint: 1 strain was *cpsO* positive, and another one was *cpsO* negative (Table 1). As the *cpsO* gene is unique to pathogenic *E. cecorum* (Borst et al., 2023), the results demonstrated that pathogenic *E. cecorum* was present on Farm 1.

**Table 1:** Genome features of *E. cecorum* isolated in this study compared to *E. cecorum* genomes from other countries.

Strain	Size (bp)	Total CDS	GC content (%)	*cpsO* gene*	Host tissue	Sample type/Country	GenBank Accession
SA3	2,320,599	2,454	36.3	(+)	Bone marrow	Clinical/USA	CP010064
CE1	2,438,959	2,636	36.2	(-)	Caecum	Nonclinical/USA	CP010059
Farm1_B1Spine	2,242,392	2,268	36.3	(+)	Vertebral lesion	Clinical/Australia	GCA_040104975.1
Farm1_B3Caecum	2,325,822	2,406	36.2	(-)	Caecum	Clinical/Australia	GCA_040104935.1
Farm1_B3SpineAB	2,243,240	2,270	36.3	(+)	Vertebral lesion	Clinical/Australia	GCA_040104955.1
Farm1_B3Spine	2,245,142	2,266	36.4	(+)	Vertebral lesion	Clinical/Australia	GCA_040104915.1
Farm1_B4Leg	2,095,370	2,150	36.7	(-)	Intertarsal joint	Clinical/Australia	GCA_040104895.1
Farm1_B5Caecum	2,182,651	2,288	36.6	(-)	Caecum	Clinical/Australia	GCA_040104875.1
Farm1_B5LegAB	2,254,097	2,279	36.3	(+)	Intertarsal joint	Clinical/Australia	GCA_040104855.1
Farm1_B5Leg	2,257,505	2,356	36.6	(-)	Intertarsal joint	Clinical/Australia	GCA_040104835.1
Farm2_B1LegC2	2,185,069	2,196	36.6	(-)	Intertarsal joint	Clinical/Australia	GCA_040104815.1
Farm2_B1SpleenC4	2,157,427	2,164	35.5	(-)	Spleen	Clinical/Australia	GCA_040104755.1
Farm2_B2LegC3	2,188,481	2,183	36.6	(-)	Intertarsal joint	Clinical/Australia	GCA_040104775.1
Farm2_B3CaecumC6	2,228,442	2,258	36.6	(-)	Caecum	Clinical/Australia	GCA_040104795.1
Farm2_B3SpleenC6	2,179,398	2,180	36.6	(-)	Spleen	Clinical/Australia	GCA_040104735.1
CIRMBP-1228	2,799,887	2,845	36.2	(+)	Joint	Clinical/France	OX346405
CIRMBP-1261	2,620,135	2,600	36.3	(+)	Joint	Clinical/France	OX346402
E1062	2,524,751	2601	36.0	(-)	Cloacal	Nonclinical/Brazil	JAKUDU000000000.1
CB-32	2,293,164	2302	36.5	(+)	Tissue sample	Clinical/Poland	GCA_947055655.1
BB-66	2,480,260	2535	36.2	(+)	Tissue sample	Clinical/Poland	GCA_947055785.1
ASM188588v1	2,316,525	2378	36.4	(-)	Caecum	Nonclinical/Mongolia	GCA_001885885.1
Chicken_16_mag_170	2,000,040	2036	36.6	(-)	Caecum	Nonclinical/Scotland	GCA_904419365.1

⁎ The *cpsO* gene was detected by PCR and/or inferred from whole genome sequences.

On Farm 2, however, none of the *E. cecorum* isolates obtained from birds were *cpsO* positive (Table 1). The *cpsO* PCR of the caecal content of the birds in this farm was positive, while *cpsO* PCR of the washed plates was negative. Therefore, pathogenic *E. cecorum* with the *cpsO* gene might be present in the second farm but failed to be isolated. This may be due to the potential presence of other inhibitory substances or competitive bacteria in the samples which hindered the growth of *E. cecorum* on the selective media used during the isolation process. Notably, *E. cecorum* isolated from the clinical sites in the second farm may be the pathogenic strain, but *cpsO*-PCR negative, as the *cps* gene alone might not be sufficient to differentiate between all pathogenic and commensal strains (Laurentie et al., 2023; Rhoads et al., 2024.) Further work, including Koch's postulates experiments, is needed to confirm whether these isolates are pathogenic.

**Figure 1:**
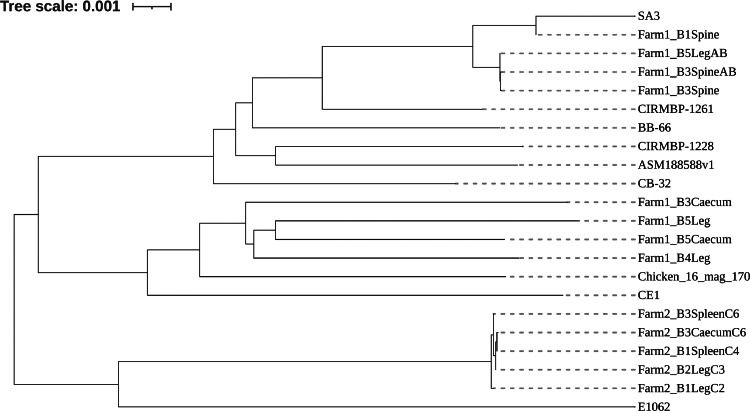
Whole genome phylogenetic analysis of *E. cecorum* strains isolated in this study compared with strains from other countries. The genome BLAST distance phylogeny method on the type strain genome server was used for the analysis.

The phylogenetic analysis using the whole genome showed the pathogenic *E. cecorum* strains in Australia had a close relationship to the clinical strain SA3 in North America, but a distant relationship to the pathogenic strains in France, and Poland (Figure 1). The pathogenic strains in Farm 1 clustered distinctly from the commensal strains. It also showed that the isolates obtained from diseased birds on Farm 2 were distant from those on Farm 1.

In summary, the isolation of the bacteria from chickens with bacterial spondylitis in Australia compared to strains from other countries revealed that the pathogenic *E. cecorum* strain was present from 1 of the 2 studied farms and had the closest relationship to the pathogenic *E. cecorum* strains in North America, while being distant from those in France and Poland. The typical pathogenic *E. cecorum* strains that were isolated from Farm 1, similar to those reported in diseased birds in other countries, with the conserved *cpsO* gene, could not be isolated from Farm 2. It is possible that the strains obtained from extraintestinal organs in this study might be pathogenic and the cause of the disease. However, further work is needed to confirm this. Comparative analysis of strains from all states in Australia will help to understand the genetic makeup of pathogenic *E. cecorum* and the development of bacterial spondylitis, aiding in reducing the economic losses incurred by farmers and the health burdens impacting broiler chickens.

## DISCLOSURES

PCS was employed by Scolexia Pty Ltd and SR was employed by Inghams Enterprises Pty Ltd. The remaining authors declare that the research was conducted in the absence of any commercial or financial relationships that could be construed as a potential conflict of interest.
